# Improved ginseng production under continuous cropping through soil health reinforcement and rhizosphere microbial manipulation with biochar: a field study of *Panax ginseng* from Northeast China

**DOI:** 10.1093/hr/uhac108

**Published:** 2022-05-17

**Authors:** Cheng Liu, Rong Xia, Man Tang, Xue Chen, Bin Zhong, Xiaoyu Liu, Rongjun Bian, Li Yang, Jufeng Zheng, Kun Cheng, Xuhui Zhang, Marios Drosos, Lianqing Li, Shengdao Shan, Stephen Joseph, Genxing Pan

**Affiliations:** Institute of Resource, Ecosystem and Environment of Agriculture, and Department of Soil Science, Nanjing Agricultural University, Nanjing 210095, Jiangsu, China; Jiangsu Collaborative Innovation Center for Solid Organic Waste Resource Utilization, Nanjing Agricultural University, 1 Weigang, Nanjing 210095, China; Institute of Resource, Ecosystem and Environment of Agriculture, and Department of Soil Science, Nanjing Agricultural University, Nanjing 210095, Jiangsu, China; Institute of Resource, Ecosystem and Environment of Agriculture, and Department of Soil Science, Nanjing Agricultural University, Nanjing 210095, Jiangsu, China; Institute of Resource, Ecosystem and Environment of Agriculture, and Department of Soil Science, Nanjing Agricultural University, Nanjing 210095, Jiangsu, China; Institute of Resource, Ecosystem and Environment of Agriculture, and Department of Soil Science, Nanjing Agricultural University, Nanjing 210095, Jiangsu, China; Institute of Resource, Ecosystem and Environment of Agriculture, and Department of Soil Science, Nanjing Agricultural University, Nanjing 210095, Jiangsu, China; Jiangsu Collaborative Innovation Center for Solid Organic Waste Resource Utilization, Nanjing Agricultural University, 1 Weigang, Nanjing 210095, China; Institute of Resource, Ecosystem and Environment of Agriculture, and Department of Soil Science, Nanjing Agricultural University, Nanjing 210095, Jiangsu, China; Jiangsu Collaborative Innovation Center for Solid Organic Waste Resource Utilization, Nanjing Agricultural University, 1 Weigang, Nanjing 210095, China; College of Chinese Medicinal Materials, Jilin Agricultural University, 28888 Xincheng Street, Changchun 130118 China; Institute of Resource, Ecosystem and Environment of Agriculture, and Department of Soil Science, Nanjing Agricultural University, Nanjing 210095, Jiangsu, China; Jiangsu Collaborative Innovation Center for Solid Organic Waste Resource Utilization, Nanjing Agricultural University, 1 Weigang, Nanjing 210095, China; Institute of Resource, Ecosystem and Environment of Agriculture, and Department of Soil Science, Nanjing Agricultural University, Nanjing 210095, Jiangsu, China; Jiangsu Collaborative Innovation Center for Solid Organic Waste Resource Utilization, Nanjing Agricultural University, 1 Weigang, Nanjing 210095, China; Institute of Resource, Ecosystem and Environment of Agriculture, and Department of Soil Science, Nanjing Agricultural University, Nanjing 210095, Jiangsu, China; Jiangsu Collaborative Innovation Center for Solid Organic Waste Resource Utilization, Nanjing Agricultural University, 1 Weigang, Nanjing 210095, China; Institute of Resource, Ecosystem and Environment of Agriculture, and Department of Soil Science, Nanjing Agricultural University, Nanjing 210095, Jiangsu, China; Jiangsu Collaborative Innovation Center for Solid Organic Waste Resource Utilization, Nanjing Agricultural University, 1 Weigang, Nanjing 210095, China; Institute of Resource, Ecosystem and Environment of Agriculture, and Department of Soil Science, Nanjing Agricultural University, Nanjing 210095, Jiangsu, China; Jiangsu Collaborative Innovation Center for Solid Organic Waste Resource Utilization, Nanjing Agricultural University, 1 Weigang, Nanjing 210095, China; Key Laboratory of Recycling and Eco-treatment of Waste Biomass of Zhejiang Province, Zhejiang University of Science and Technology, Hangzhou 310023, China; Institute of Resource, Ecosystem and Environment of Agriculture, and Department of Soil Science, Nanjing Agricultural University, Nanjing 210095, Jiangsu, China; School of Materials Science and Engineering, University of New South Wales, Sydney, New South Wales 2052, Australia; Institute of Resource, Ecosystem and Environment of Agriculture, and Department of Soil Science, Nanjing Agricultural University, Nanjing 210095, Jiangsu, China; Jiangsu Collaborative Innovation Center for Solid Organic Waste Resource Utilization, Nanjing Agricultural University, 1 Weigang, Nanjing 210095, China

## Abstract

The production of ginseng, an important Chinese medicine crop, has been increasingly challenged by soil degradation and pathogenic disease under continuous cropping in Northeast China. In a field experiment, an Alfisol garden continuously cropped with Chinese ginseng (*Panax ginseng* C. A. Meyer) was treated with soil amendment at 20 t ha^−1^ with maize (MB) and wood (WB) biochar, respectively, compared to conventional manure compost (MC). Two years after the amendment, the rooted topsoil and ginseng plants were sampled. The changes in soil fertility and health, particularly in the soil microbial community and root disease incidence, and in ginseng growth and quality were portrayed using soil physico-chemical assays, biochemical assays of extracellular enzyme activities and gene sequencing assays as well as ginsenoside assays. Topsoil fertility was improved by 23% and 39%, ginseng root biomass increased by 25% and 27%, and root quality improved by 6% and 18% with WB and MB, respectively, compared to MC. In the ginseng rhizosphere, fungal abundance increased by 96% and 384%, with a significant and insignificant increase in bacterial abundance, respectively, under WB and MB. Specifically, the abundance of *Fusarium* spp. was significantly reduced by 19–35%, while that of *Burkholderia* spp. increased by folds under biochar amendments over MC. Relevantly, there was a significant decrease in the abundance proportion of pathotrophic fungi but a great increase in that of arbuscular mycorrhizal fungi, along with an enhanced microbial community network complexity, especially fungal community complexity, under biochar amendments. Thus, biochar, particularly from maize residue, could promote ginseng quality production while enhancing soil health and ecological services, including carbon sequestration, in continuously cropped fields.

## Graphical Abstract

**Graphical Abstract:**
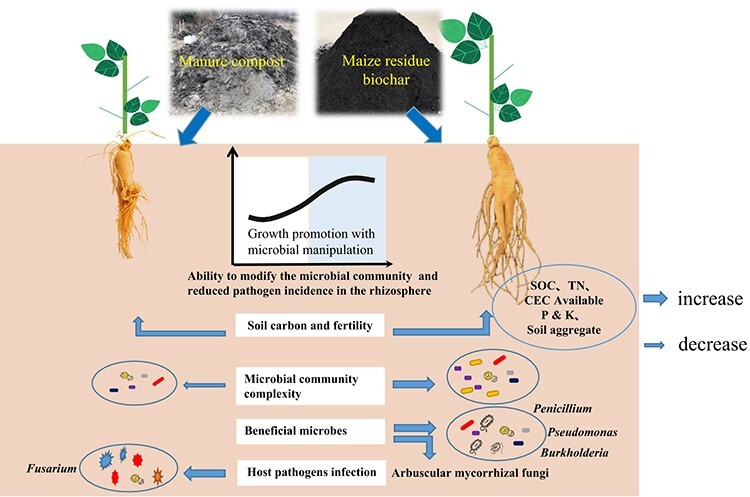

## Introduction

Ginseng (*P. ginseng* C. A. Meyer), an important root tuber crop widely used in biochemical pharmacology [1], is traditionally grown in deep, soft and well-drained soils rich in humus and key nutrients such as phosphorus [2]. This kind of ginseng has been increasingly cultivated under continuous cropping in Northeast China [3], leading to serious soil degradation with organic matter depletion, soil compaction and acidification and disordered soil food web systems [4]. Consequently, soil health has often significantly declined with root allelopathy [5] and soil-borne diseases such as root rot and rusty roots [6] in continuously cropped ginseng plants. Such root diseases were related to the activities of cell wall degrading enzymes (CWDEs) [7], which are potentially produced by soil-borne pathogens attacking organic matter for nutrient competition [8]. Thus, the functions of these degraded ginseng soils related to organic carbon storage, N and P availability and biodiversity were greatly damaged [9]. These negative changes caused a significant decline in root yield and the functional quality of ginseng, challenging farmers’ revenue [10]. Local ginseng farmers used topsoil replacement or compost amendment from crop residue and animal manure, together with soil fumigants and/or fungicides [2]. Unfortunately, neither soil health nor ginseng production has been well recovered, raising an emerging issue of pesticide residue impacts on ginseng plants and on the soil microbiome.

Soil health in agricultural systems has been increasingly considered vital for food production and thus global sustainability [11]. Generally, soil health represents the continuing capacity of a soil to function as a vital living ecosystem that sustains plants, animals and humans with the safe provisioning of multiple ecosystem services [12]. Global actions were urged to protect soil health and food safety by manipulating the sustainable management of the soil for food production, supply chains for consumption and waste treatment, and the restoration of degraded ecosystems [13] within the context of the United Nations Sustainable Development Goals of Earth governance [14]. As widely recognized, soil organic matter (C as an element) acts as a key driver of soil health [15, 16]. Thus, reinforcing soil organic matter through residue management or organic amendments has been in global demand to sustain soil health and reverse soil degradation for global food security and climate stabilization [17, 18].

Soil health has been characterized and assessed with biophysical, biochemical and biological indicators [11]. Soil biological health has been increasingly given priority for enhancing soil functions to improve nutrient availability, crop protection, stress resilience and plant disease resistance in agriculture [19]. For this, soil health could be conceived as the vitality of soil in sustaining the socioecological functions of its enfolding land, with a biological focus on land- or ecosystem-specific behavior [20]. However, biological health has been poorly evaluated and interpreted for the safe provisioning of ecosystem services [12]. As soil biota are closely linked to soil structure/aggregates for carbon and space allocation [21], whether and how soil organic matter reinforcement could be taken as an ecological engineering measure to cope with the improvement of soil biological health have been poorly understood.

Being massively generated in the food supply chain, biowastes or organic residues pose potential ecological and environmental risks associated with toxic metals and antibiotic resistance genes, limiting their value in soil organic matter reinforcement [22]. Biochar, which is thermos converted from biowastes, has been globally advocated for enhancing soil carbon sequestration [23, 24] and soil functions for nutrient availability and fertility [25], crop production [26] and decontamination [27] in agriculture. Biochar amendments could lead to biophysical [28], biochemical [29] and biological [30] improvements in agricultural soils, contributing to improved soil health through increased microbial diversity and metabolic activity [30–32]. Such biochar-induced soil health improvement contributed to the increased microbial diversity and activity in the biochar-amended rhizosphere, a potential mechanism of biochar-induced system resistance (ISR) [33] with suppression of soil-borne plant diseases [34]. These effects were shown potentially through reduced colonization of pathogens in the soil and enhanced growth promoting or biocontrolling beneficial microorganisms [33, 35]. Yet, the biochar addition effect on improving soil biological health and root disease resistance in ginseng production under continuous cropping remains unclear.

In this study, we hypothesize that ginseng root production could be improved by reinforcing soil fertility/health and, in turn, microbial manipulation through biophysical, biochemical and biological improvements in continuously cropped fields with biochar amendment. Biophysical improvements could help shift the soil microbiome with enhanced beneficial microorganisms against soil-borne pathogens in the rhizosphere. To test this hypothesis, a field experiment with biochar soil amendment was conducted on a typical ginseng farm under ginseng growth for 3 years in a ginseng production area in Jilin, Northeast China. Changes in soil health and plant production/quality were explored with soil fertility, biochemical, microbial sequencing and ginsenoside assays. This study aimed to develop an ecological measure with biochar instead of conventional manure compost for improving soil biological health and ginseng quality production under continuous cropping stresses.

## Results

### Soil properties, ginseng growth and root production

The edaphic properties of bulk topsoil (0–15 cm) sampled at 2 years following the amendment are listed in Table 1. Most of the soil properties analyzed were significantly improved under biochar additions over MC. Compared to MC, soil pH was elevated by 0.2–0.3 units and EC was reduced by 50–60% under both WB and MB, while CEC remained unchanged under WB but increased by 14% under MB. In particular, SOC was enhanced by 15% and 45%, while available P was enhanced by 79% to 134% under WB and MB, respectively, relative to MC. The soil C/N ratio increased insignificantly under WB (12.9) and significantly under MB (16.1) over MC (12.4). In contrast, the mean weight diameter (MWD) of water-stable aggregates, known as soil aggregate stability, was increased by 14% under WB and by 9% under MB compared to MC. On average, there was an overall improvement in soil fertility by 23% under WB and by 39% under MB over MC.

**Table 1:** Properties of topsoil (0–15 cm) in harvest at 2 years following amendment at 20 t ha^−1^ in the continuously cropped ginseng farm

Treatment	pH (H_2_O)	B.D. (g cm^−3^)	SOC (g kg^−1^)	Total N (g kg^−1^)	Available P (mg kg^−1^)	Available K (mg kg^−1^)	CEC (cmol kg^−1^)	Moisture (%)
MC	4.45 ± 0.11b	**1.09 ± 0.02a**	18.79 ± 1.15c	1.38 ± 0.16a	17.90 ± 2.31c	341.1 ± 12.97c	22.90 ± 0.80b	11.03 ± 0.53c
WB	**4.78 ± 0.09a**	1.02 ± 0.01b	21.72 ± 1.32b	1.43 ± 0.05a	**32.09 ± 1.81b**	**373.7 ± 4.42b**	23.04 ± 1.87b	**12.90 ± 0.91b**
MB	**4.65 ± 0.04a**	0.97 ± 0.06b	**27.30 ± 1.21a**	1.44 ± 0.29a	**41.94 ± 1.97a**	**490.7 ± 4.59a**	**26.22 ± 1.04a**	**15.83 ± 1.02a**
Treatment	EC (μs cm^−1^)	MBC (mg kg^−1^)	MBN (mg kg^−1^)	MBC/MBN	Macroaggregates (250–2000 μm)	Microaggregates (53–250 μm)	Fine particles (<53 μm)	MWD (μm)
MC	74.83 ± 9.10a	98.86 ± 3.09b	6.25 ± 0.40b	**15.84 ± 0.65a**	31.94 ± 0.77b	**17.00 ± 1.04a**	51.06 ± 1.65a	398.6 ± 9.40b
WB	**23.00 ± 1.08c**	**114.09 ± 1.70a**	9.27 ± 1.48ab	12.52 ± 2.00ab	**38.21 ± 3.74a**	7.62 ± 0.60c	54.17 ± 3.59a	**455.8 ± 40.8a**
MB	**35.10 ± 1.57b**	**114.63 ± 8.16a**	11.05 ± 3.33a	10.84 ± 2.35b	**35.71 ± 0.72a**	11.92 ± 2.91b	52.37 ± 2.89a	**433.7 ± 8.22a**

B.D., bulk density; SOC, soil organic carbon; CEC, cation exchange capacity. MC, manure compost; WB, wood biochar; MB, maize biochar. Different letters in a single block column indicate significant differences between treatments at *p* < 0.05.

Compared to MC, soil MBC was enhanced by 15%, while MBN was enhanced by 48% and 77% under WB and MB, respectively. Relevantly, the portion of MBN to total N, also known as the microbial quotient of N, was significantly increased by 34% under WB and by 59% under MB compared to MC. In contrast to the soil, the microbial C/N ratio was reduced by 21% and 32% under WB and MB, respectively, over MC.

**Figure 1:**
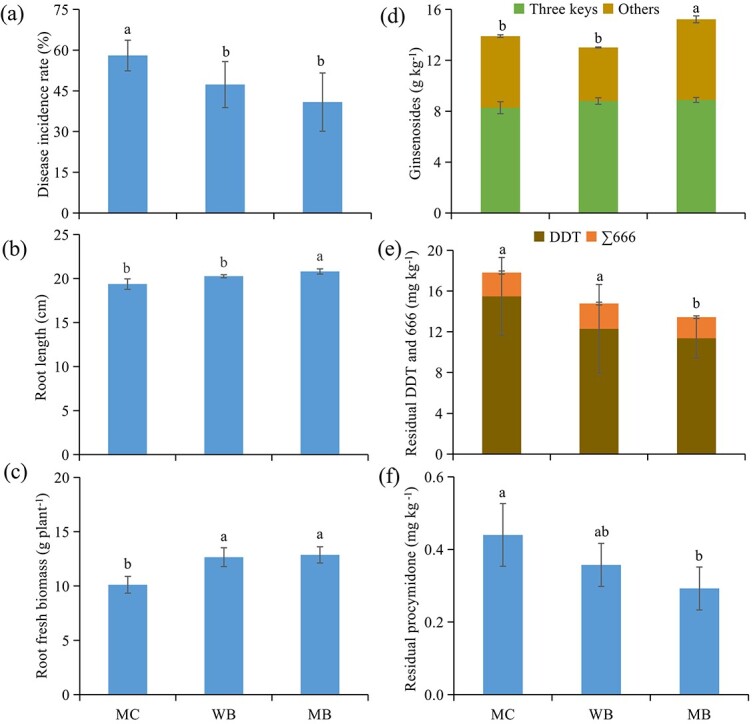
Growth parameters and quality of ginseng. The disease incidence rate (a), root size (b), root fresh biomass (c), contents of ginsenosides of the key ginsenosides (Rg1 + Rb1 + Re) and the others (d), of residual toxic pesticides of DDT and 666 (e), and residual procymidone (f), of ginseng roots harvested 2 years following amendment at 20 t ha^−1^. MC, manure compost; WB, wood biochar; MB, maize biochar. Different letters over the same color bars in a single bar graph represent a significant difference among the treatments at *p* < 0.05.

The key parameters of ginseng root growth measured at harvest are organized in Fig. 1a, b, c, while the growth traits of the transplanted ginseng plants observed at harvest are listed in Table S3. Although the change in growth traits with biochar addition was variable across the parameters, a general improvement was observed by 10% under WB and by 17% under MB over the MC. Hereby, the ginseng root size was significantly but slightly (5–7%) increased under biochar addition compared to MC. However, the survival rate, plant density and root build-up all increased, but the root rot incidence decreased to the extent of 20–27% under WB and 27–58% under MB over MC. Such changes could be generally viewed in the field (Fig. S4). Relevantly, the yield of ginseng roots harvested was increased by 58% under WB (119.5 g m^−2^) and by one-fold under MB (150.8 g m^−2^) compared to that under MC (75.9 g m^−2^). On average, plant production was improved by 23% and 42% under WB and MB, respectively, relative to MC.

Original data of the contents of ginsenosides and toxic residual pesticides of harvested ginseng roots are listed in Tables S4 and S5, respectively. With the health concern of ginseng root quality, changes in the total content of the key monomers of ginsenosides and residual pesticides are plotted in Fig. 1. Compared to MC, the total ginsenoside content was unchanged under WB but significantly increased by 10% under MB, while the cumuli content of Rb1, Re and Rg3, the three key components regulated for ginseng in EU, USA and China, was relatively higher under biochar addition than under MC. Under MB than under WB, the total ginsenoside content and nonkey monomer contents of Rf, Rc, Rb2, Rb3, Rd and Rh2 were higher (Table S4). Furthermore, in the harvested samples of ginseng roots, residual pesticides p,p’-DDE and pentachloro-nitrobenzene were undetected, while the contents of α-666 and δ-666 were unchanged under WB but significantly decreased by over 10% under MB compared to MC (Table S5). Meanwhile, the content of procymidone was significantly reduced by 18% under WB and by 33% under MB over MC. Weighted by the beneficial ginsenosides and unwanted residual pesticides as per Equation (3), the overall root quality production was improved by 55% and 116%, respectively, under WB and MB over MC.

### Soil extracellular enzyme activity

Data on soil extracellular enzyme activities (EEAs) under the treatments are given in Table 2. Under WB and MB compared to MC, EEA was increased insignificantly for α-Glucosidase and polyphenol oxidase but significantly for sucrose, urease and peroxidase, although it was decreased for acid phosphatase. Under biochar treatments, urease activity was increased by nearly 5-fold. Related to cell wall degradation, β-Glucosidase, cellobiohydrolase and N-acetyl-glucosaminidase showed reduced activity by 10–50% under biochar treatments over the MC. An overall improvement of EEAs was estimated by 54% under WB and 76% under MB over MC. Overall, biochemical properties were improved by 7% under WB and 22% under MB compared to MC.

**Table 2:** Soil extracellular enzyme activities of rooted topsoil (0–15 cm) in harvest at 2 years following amendment at 20 t ha^−1^ in the continuously cropped ginseng farm

Treat ment	α-Glucosidase (nmol g^−1^ h^−1^)	β-Glucosidase (nmol g^−1^ h^−1^)	β-Xylosidase (nmol g^−1^ h^−1^)	Cellobiohydrolase (nmol g^−1^ h^−1^)	Polyphenol oxidase (μmol g^−1^ h^−1^)	Sucrase (mg g^−1^ d^−1^)
MC	5.50 ± 0.22a	**136.84 ± 1.76a**	19.49 ± 1.52b	**25.63 ± 1.19a**	0.28 ± 0.01a	25.78 ± 1.89b
WB	6.61 ± 0.98a	113.17 ± 1.95c	16.66 ± 1.23c	**22.93 ± 0.79b**	0.29 ± 0.01a	**33.82 ± 1.23a**
MB	6.98 ± 0.95a	123.37 ± 1.19b	**24.61 ± 1.22a**	20.08 ± 1.37c	0.29 ± 0.00a	**36.35 ± 1.15a**
**Treat ment**	**Urease** **(mg g^−1^ d^−1^)**	**NAG** **(nmol g^−1^ h^−1^)**	**Acid phosphatase** **(nmol g^−1^ h^−1^) **	**Sulfatase** **(nmol g^−1^ h^−1^)**	**Peroxidase** **(μmol g^−1^ h^−1^)**	**Normalized** **enzyme activity**
MC	0.03 ± 0.00b	**29.72 ± 2.45a**	**175.76 ± 1.52a**	8.70 ± 0.98b	4.73 ± 0.13c	0.10 ± 0.00b
WB	**0.17 ± 0.02a**	17.90 ± 1.75b	158.56 ± 0.60c	7.99 ± 1.59b	**5.60 ± 0.28b**	0.11 ± 0.00b
MB	**0.18 ± 0.01a**	14.80 ± 1.94b	167.10 ± 0.75b	**19.30 ± 1.63a**	**6.29 ± 0.10a**	**0.12 ± 0.00a**

NAG, N-acetyl-glucosaminidase; MC, manure compost; WB, wood biochar; MB, maize biochar. Different letters in a single column indicate significant differences between treatments at *p* < 0.05.

**Figure 2:**
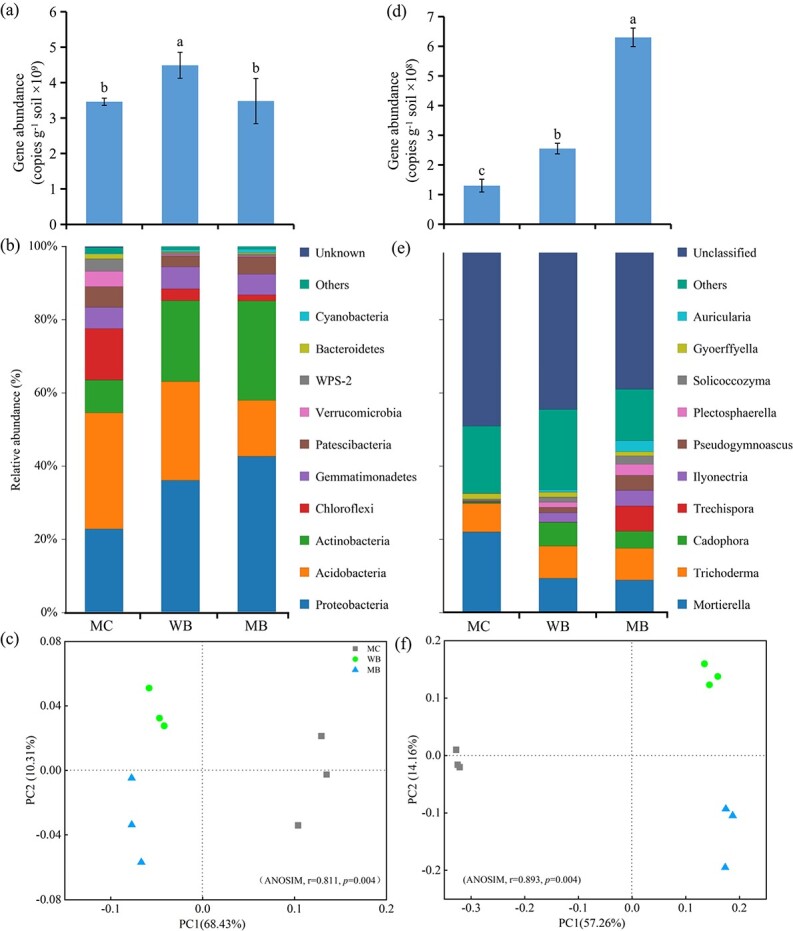
Gene abundance (a, d) and top 10 phyla composition (b, e) of bacterial and fungal community and principal coordinate analysis (PCoA) ordinations of bacterial (c) and fungal (f) community composition based on unweighted UniFrac distance metric of rhizosphere soil sampled at harvest 2 years following soil amendment at 20 t ha^−1^. MC, manure compost; WB, wood biochar; MB, maize biochar. Differences in bacterial and fungal beta diversity were determined by analysis of molecular variance (ANOSIM). Different letters above the bars in a single bar graph indicate significant differences among the treatments at *p* < 0.05.

### Soil microbial abundance and community structure

Data on the total gene abundance and community structure at the phylum level of bacteria and fungi in rhizosphere soil sampled at ginseng harvest are organized in Fig. 2. Under WB and MB over MC, the gene abundance of bacteria was increased (by 30%) and unchanged, while that of fungi increased significantly and strongly by 96% and 385%, respectively. The sequences obtained across treatments were classified into 21 phyla for bacteria and 13 phyla for fungi. The top 10 bacterial phyla were dominated by *Proteobacteria* (23–43%), *Acidobacteria* (15–32%) and *Actinobacteria* (9–27%), followed by *Gemmatimonadetes* (5–6%) and *Patesci-bacteria* (3–6%), in addition to others in smaller abundance (<4%). The *Proteobacteria* and *Actinobacteria* increased, while those of *Acidobacteria, Chloroflexi* and *Verrucomicrobia* decreased markedly with biochar addition compared to MC. Meanwhile, the top 10 fungal phyla were dominated by *Mortierella* (9–22%), followed by *Trichoderma* (8–9%), along with others in a proportion between 0.02–7.0%. Hereby, the proportion of *Mortierella* decreased, while that of *Cadophora*, *Ilyonectria*, *Pseudogymnoascus* and *Plectosphaerella* increased markedly with biochar amendments over MC. At the phylum level, α-diversity as per the Shannon index of bacteria was reduced under WB and MB, while that of fungi significantly increased under WB and MB by 44% and 104%, respectively, compared to MC.

Calculated based on the rarefied sequences, richness (Chao1) and Shannon diversity of both the bacterial and fungal communities are given in Table S6. There was a significant (*p* < 0.05) increase in richness (Chao1) and an insignificant increase in Shannon diversity of both bacterial and fungal communities with biochar treatment over MC. By a principal coordinate analysis (PCoA) (Fig. 2), the soil microbial community was clearly separated among the treatments, by PC1 between biochar treatment and the MC, and by PC2 between WB and MB (ANOSIM, *p* < 0.001), regardless of bacterial or fungi.

**Figure 3:**
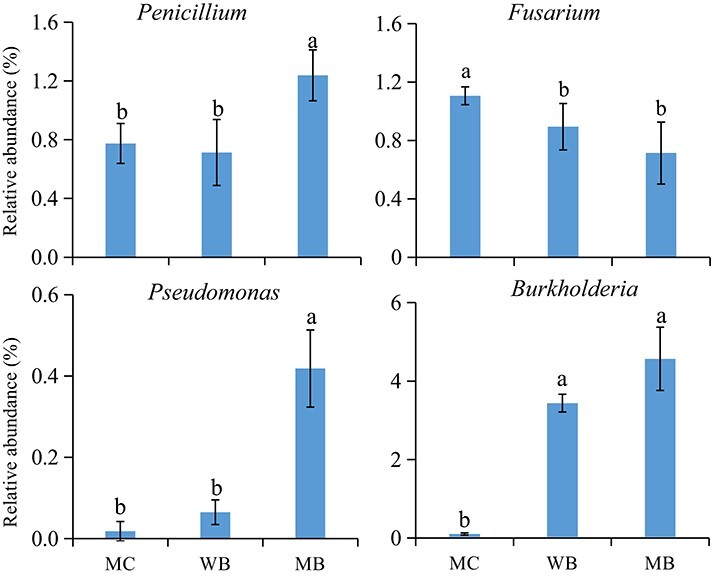
Relative abundance of beneficial genus of *Penicillium*, *Pseudomonas* and *Burkholderia*, and the potential pathogen fungus *Fusarium* in the fungal community of rhizosphere soil sampled in the harvest at 2 years following amendment at 20 t ha^−1^. MC, manure compost; WB, wood biochar; MB, maize biochar. Different letters above the bars in a single bar graph indicate significant differences among the treatments at *p* < 0.05.

At the genus level, the bacterial community was composed of 26 genera. The relative abundance of the 16 main genera, in an individual proportion > 0.1%, was higher under biochar amendments than under MC. For beneficial bacterial species, compared to MC, the relative abundance of *Burkholderia* was increased by a fold under both WB and MB, while that of *Penicillium and Pseudomonas* significantly increased under MB only (by over 50%) (Fig. 3). For the fungal taxa, the relative abundance of *Fusarium*, a potential pathogenic fungus, was significantly decreased by 19% and 35% under WB and MB, respectively, over MC.

**Figure 4:**
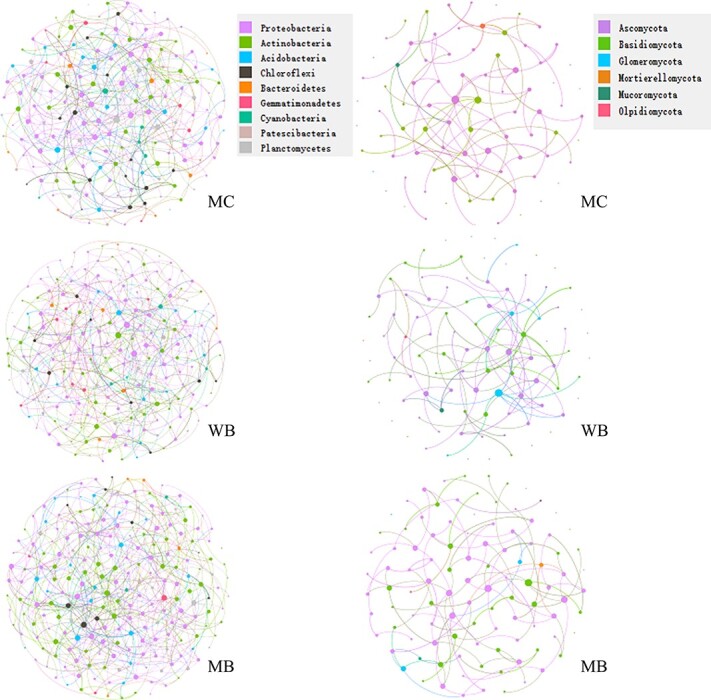
Bacterial (left) and fungal (right) co-occurrence networks colored by their phyla, based on Spearman’s correlations, of rhizosphere soil sampled in the harvest at 2 years following amendment at 20 t ha^−1^. The size of each node is proportional to the number of connections (degree), and the thickness of each edge is proportional to the value of Spearman’s correlation coefficients. The red edges denote negative interactions while the blue edges denote positive interactions between two nodes. MC, manure compost; WB, wood biochar; MB, maize biochar.

To gain further insight into the differences in microbial communities among the treatments, co-occurrence networks were constructed to visualize the relationships among the OTUs of both bacteria and fungi (Fig. 4). The data of the number of nodes and edges, the average path length, the average clustering coefficient and modularity under the treatments are given in Table S8. Obviously, the networks of bacterial phyla looked significantly different from those of fungal phyla among the treatments. In general, the bacterial networks had higher numbers of nodes and edges under biochar amendments than under MC. However, the average path length, average clustering coefficient and modularity under WB and the clustering coefficient under MB of bacterial phyla were higher than those under MC. For fungal networks, the number of nodes and edges, the average path length, the average clustering coefficient and the modularity of fungal phyla were all higher under WB and MB than under MC.

**Table 3:** Percentages of reads assigned to different fungal guilds of ginseng rhizosphere sampled in harvest at 2 years following amendment at 20 t ha^−1^ in the continuously cropped ginseng farm

Treatment	Symbio -trophic	Sapro -tropic	Patho -tropic	Endophyte	Arbuscular Mycorrhizal	Dung saprotroph	Leaf saprotroph	Wood saprotroph	Undefined saprotroph	Fungal parasite
MC	69.1 ± 30.7ab	**27.5 ± 32.1a**	**0.87 ± 0.15a**	63.6 ± 30.5a	0.41 ± 0.63b	27.4 ± 32.0a	0.04 ± 0.06a	*n.d.* c	2.87 ± 0.99a	**0.58 ± 1.01a**
WB	50.7 ± 21.8b	**49.2 ± 21.8a**	0.02 ± 0.01b	43.0 ± 18.5a	**6.88 ± 2.68a**	**0.40 ± 0.14b**	0.49 ± 0.49a	48.3 ± 22.3a	0.07 ± 0.02b	0.02 ± 0.01b
MB	**98.1 ± 1.19a**	1.0 ± 0.74b	0.47 ± 0.15b	86.5 ± 6.40a	**10.4 ± 4.99a**	**0.60 ± 0.53b**	0.03 ± 0.05a	0.38 ± 0.28b	0.36 ± 0.32b	0.47 ± 0.15a

MC, manure compost; WB, wood biochar; MB, maize biochar. *n.d.*, not detected. Different letters in a single column indicate significant differences between treatments at *p* < 0.05.

### Bacterial and fungal communities with predicted functions

The changes in the functional traits of the bacterial community on the KEGG pathway (KO tier 2) at the DNA level of the rooted soil between biochar and manure application are plotted in Fig. S8. Among the second-tier functional categories, the abundance of functional traits associated with “Xenobiotics”, “Metabolism of other amino acids”, “Amino acid metabolism”, “Membrane transport” and “Cellular community-prokaryotes” were significantly higher under biochar addition than under conventional manure. In parallel, the relative abundances of functions with “Glycan biosynthesis and metabolism”, “Biosynthesis of other secondary metabolites”, “Carbohydrate metabolism”, “Energy metabolism” and “Translation” were slightly but significantly lower.

The relative abundance of fungal sequences assigned to functional guilds with ecological significance, together with seven guilds involved in the three trophic categories, is presented in Table 3. By trophic mode, the abundance proportion of the “pathotrophic” fungal group was very significantly (*p* < 0.01) decreased under WB and MB over MC. In contrast, the proportion of “symbiotrophic” was significantly (*p* < 0.01) lower, while that of “saprotrophic” was significantly but greatly higher under WB than MC. Under MB, “saprotrophic” almost disappeared, and “symbiotrophic” predominated the relative proportion. Over MC, the proportions of both arbuscular mycorrhizal and woody saprotrophs increased, while those of dung and undefined saprotrophs decreased significantly (*p* < 0.05) with WB and MB. In addition, the proportion of fungal parasites was decreased insignificantly under WB but significantly and greatly under MB over MC.

## Discussion

### Ginseng production and soil health synergistically improved with biochar

Soil health has been increasingly concerned with the capacity to provide ecosystem functions and services, including the provisioning of nutrients, SOC and moisture, as well as biodiversity [52]. With continuous cropping of ginseng, the acid Alfisol originally under forest had been degraded with sharp SOC decline, soil acidification and poor availability of key nutrients such as P and K, along with incidence of soil-borne pathogenic diseases [24]. Against this obstacle in ginseng farming, manure compost has been conventionally used [53]. In this study, compared to manure use, biochar addition exerted a significant positive change in overall fertility by over 20% (available P elevated by over 80% and EC reduced by 50%, in particular) (Table 1). Estimated with Equation (1) using the dataset including soil biochemical and biological properties (Table S7), basic soil health, or soil fertility, was improved significantly by 9% under WB but 18% under MB over the MC. Therefore, the addition of biochar rather than manure could be used to improve the soil fertility of ginseng fields under continuous cropping stress.

Moreover, biochar addition could create a more or less synergistic improvement of soil functions, which is the key to soil health [20]. In this study, SOC storage was unchanged following MC addition but increased by 2.5 t ha^−1^ and 9.0 t ha^−1^ following WB and MB addition, respectively. The available pools of P and K, microbial biomass and enzyme activities were significantly but moderately to greatly alleviated, while soil acidity and salinity were markedly alleviated (Table 1, Table 2) following biochar amendment. As per the data in Table S6, microbial diversity was coincidently enhanced under biochar addition over MC. In line with these changes, ginseng plant growth (Table S3, Fig. 1) and root quality, weighted by beneficial ginsenosides and toxic residual pesticides (Table S9), were all improved by 10–20% under biochar addition over MC. While potential tradeoffs between soil ecosystem services were seriously concerned with biochar soil amendments in agriculture [54], this study showed a more or less synergistic improvement of soil functions of SOC sequestration, nutrient availability and microbial growth/activity, as well as reducing the stress of soil salinity and acidity by biochar in the continuously cropped field.

Notably, biochar induced positive changes in soil health related to the survival of plant seedlings under continuous cropping stress in this study. The survival of transplanted ginseng seedlings, the root build-up (Fig. 1) and thus the ginseng root yield (Table S9) were all promoted by approximately 20% under WB and over 50% under MB relative to MC. Such productivity improvement seemed much greater than a general increase in plant productivity by approximately 10% [26] and in plant roots by 27% [55] in global experiments with biochar soil amendments. The relative changes by over 30% to fold in the microbial quotient of N (Table 1), EEAs (particularly those of CWDEs) (Table 2) and microbial gene abundance and diversity, particularly of fungi (Fig. 2), were parallel to the ginseng production improvement under biochar compared to MC. This revealed a more profound role of biochar on soil biological health than on physicochemical health. Lehmann et al. [12] stated that management effects on soil health related to plant growth and food production should be revisited biologically, particularly in agriculture.

Ginseng root yield has been seriously impacted by the low survival rate of transplanted seedlings due to root disease incidence under continuous cropping [5]. Biochar is already known as a strong absorbent of organic compounds in soil [27] and thus has a significant capacity to absorb root exudates [34]. Accordingly, biochar could help immobilize the allergy compounds released by ginseng roots, alleviating the root rot disease incidence (by 18%–30%, Fig. 1a) following addition in this study. As reported by Asao et al. [56], the use of activated carbon led to a great yield increase in a tuber food crop stressed by continuous cropping, potentially through immobilization of a variety of alleles (mostly toxic organic acids) accumulated with the plant roots. The change in both survival and root rot incidence could be further linked to the improvement of soil biological health based on the microbial community and enzyme activities (also see Section below). Following Graber et al. [57] on the biochar-induced potential system acquired resistance (SAR), this study highlighted an inspiring role of biochar addition in improving ginseng production by potentially improving biocontrol against continuous cropping stresses. Therefore, biochar’s role in improving soil biological health [12] and thus plant defense [33] should be prioritized for continuously cropped soils.

Regarded by ginseng quality (Fig. 1), the content of total ginsenosides in the harvested ginseng roots was either unaffected or slightly increased under biochar addition. However, the additive content of the three key components of Rb1, Re and Rg3, which are regulated for ginseng in the EU, USA and China, was relatively higher under both WB and MB than under MC. Relevant to this was the reduced contents of residual pesticides in the ginseng roots under biochar treatments relative to MC (Fig. 1), although the toxic pesticides 666 and DDT were abandoned two decades ago, and procymidone was used for plant protection of ginseng consistently across the amendment treatments. Consequently, a weighted assessment revealed a significant improvement of the overall ginseng quality slightly (by 6%) under WB and moderately (by 18%) under MB over MC (Section 3.1). Interestingly, this improvement was more or less synergic with the soil fertility (or basic soil health) and plant/root growth improvement mentioned above. As shown in a field trial by Eo et al. [58], the use of vermi-compost in ginseng fields failed to improve ginseng quality despite an increase in root growth.

Concerning ginsenosides as the key commercial value, a quality production (PG) value of ginseng farming estimated using Equation (3) was promoted by 1.5 ~ 2.2-fold with biochar addition over the conventional manure (Table S9). The decreased plant disease incidence rather than root growth (Fig. 1) contributed to this economic production increase with biochar addition was much greater than the reported mean increase in crop productivity by 11% [26, 54]. Improvement of nutrient use efficiency with biochar was seen by 12–13% for N in rice paddies [35, 59] and by 25–80% for P in upland wheat and maize [60]. This could shed light on the potential of biochar in improving production and the economic efficiency of fertilizer inputs for food crops with specific function values, which are often stressed by continuous cropping. The greatly improved economic efficiency for nutrient inputs was coincident with the higher microbial N quotient (Table 1) and urease activity (Table 2), as well as the bacterial abundance of amino-acid metabolism (Fig. S8) and greatly elevated available pool of P and K (Table 1). As suggested in previous works, such nutrient efficiency improvement was linked to enhanced microbial transformation of N [61] and soil nutrient availability [25]. Therefore, biochar addition could strengthen the biological role in mediating nutrient supply to plant roots, improving the nutrient efficiency in agriculture, even though it had not been thoroughly addressed.

Following Lehmann et al. [12] and mainly Janzen et al. [20], soil health in ginseng farming could be mainly addressed for major soil functions, such as maintaining carbon storage, provisioning available nutrients of N, P and K as well as moisture, preserving microbial biomass and diversity and mediating plant growth conditions against soil acidification and salinity as well as supporting crop production. Synthesizing the data obtained in this study, the relative improvement of soil health in the ginseng farm following biochar addition is quantified and plotted in Fig. S9. Herein, the overall soil health was greatly improved under the biochar treatments, generally synergic among the major functions. Importantly, the most significant improvement was found for quality production, a key factor for soil health concerned as a land- or system-relevant metaphor [20]. In synergy with other key functions, the overall soil health improvement was 27% and 50% under WB and MB, respectively, relative to MC. Therefore, it could be proposed that the addition of biochar from agro-wastes to replace conventional manure could be an approach to boost synergic soil health and ginseng production in continuously cropped fields. As per Filchev and Bouma [62], such an approach could significantly contribute to the UN Sustainable Development Goals (SDGs), particularly farmers’ revenue through increased production and climate stabilization through carbon sequestered in soil.

### Microbial manipulation with the communities shifted with biochar

Continuously cropped plants impacted soil health and induced soil-borne plant diseases, which again altered soil biotic and abiotic performance, disrupted the balance of microbial community structure and accumulated pathogens in their rhizosphere [63, 64]. In the soil previously under forest, SOC and microbial biomass were already depleted following 3 years of continuous ginseng cropping. Along with a moderate increase in microbial biomass, biochar addition led to a great increase (by 1–4-fold) in fungal gene abundances despite a slight increase in bacterial gene abundance over the MC. Wu et al. [65] reported an increase in gene abundance of both soil bacteria and fungi by 1–2-fold following rice hull-derived biochar addition in a continuous cropped field with *Radix pseudostellariae*. In agricultural soils under cereal crops, biochar addition often caused an increase in microbial gene abundance rather than fungal abundance [35, 66]. In this study, the great increase in fungal abundance was in agreement with the marked changes in soil C/N, soil moisture and ginseng root production despite a pH increase under biochar treatments over the MC (Table 1). As shown in previous studies [67, 68], diverse microbes in soil exert complicated responses to biochar-induced changes in nutrient availability and soil moisture as well as ecological niches. Herein, fungi’s sharper response than bacteria could be attributed partly to changes in soil-root interfaces [69], with increased root growth and potential release of root-derived organic matter (Table 1) under biochar treatments.

For bacteria, biochar addition slightly increased gene richness but unchanged the diversity (Table S6) while shifting its community structure (Fig. 2). Hereby, a great increase in the relative abundance of *Proteobacteria* and *Actinobacteria* was in line with a great decrease in the relative abundance of *Acidobacteria* and *Chloroflexi*. This could represent the changes in carbon storage and soil reaction under biochar treatments over the MC. Jenkins et al. [70] reported a positive response by *Proteobacteria* to soil labile OC pools [24] but a negative response by *Acidobacteria* to dissolvable organic carbon (DOC) and pH [71]. In the degraded soils, a higher relative abundance was found for *Acidobacteria* than in the healthy soils, indicating that the microbial community diversity in the degraded soils differed from that in the healthy soils. In particular, a significant increase in the relative abundance of *Actinobacteria,* known to form a biological barrier layer around plant roots to prevent soil-borne diseases and enhance the survival of young seedlings [72], agreed with the biochar effect on the biocontrol of ginseng plants mentioned above.

Furthermore, the higher relative abundance of beneficial functional traits but lower functional traits in response to stress suggested improved bacterial metabolic activity for nutrient biotransformation, toxicant degradation and energy use efficiency in the biochar-treated soil. Similarly, Lu et al. [35] reported positive changes in the relative abundance of bacteria with beneficial functional traits in a rice paddy over the years following one biochar addition. In addition, the relative abundances of the beneficial bacterial genera *Burkholderia* and *Pseudomonas,* belonging to the *Bacillus* family, were found to sharply increase with biochar addition (Fig. 3). As these species are well known for plant growth promotion and biocontrol [73], our study demonstrated beneficial changes in the bacterial community in the stressed soil with biochar.

Unlike bacteria, the gene richness and Shannon diversity of fungi at the phylum level significantly increased under biochar addition over MC (Fig. S5). There was a profound change in fungal community composition at 2 years following biochar addition (Fig. 2). As shown with Pearson’s correlation analysis (Fig. S7), most of the soil chemical variables were significantly and positively correlated with fungal community parameters but slightly and negatively correlated with bacterial community parameters. Generally, a more complex co-occurrence network could be developed following biochar addition to soil [74]. Similarly, in the present study, the nodes, edges, average path length and fungal network modularity all increased with biochar addition compared with MC (Fig. 4; Table S8). High complexity networks normally tended to have greater stability due to network buffering, which represented improved pore structure and size as per the change of soil aggregate mean weight diameter (Table 1). Thus, the whole fungal system was manipulated with improved interaction/cooperation between fungal families to maintain functions for the healthy growth of ginseng. Consequently, core co-occurrence networks exhibited strong resistance as pathogenic fungi increased under biochar-amended soil, while manure compost-amended soil networks were prone to be affected by slight disturbances of pathogens. The relative abundance of *Moritierella,* a major phylum next to *Auricularia* (a common fungal phylum under forest in the area) of the fungal community in the stressed soil, was greatly (by >50%) decreased with a relevant increase in *Cadophora, Pseudogymnoascus, Ilyonectria* and *Plectosphaerella* under biochar treatments. *Moritierella* was shown to be sensitive to soil acidification in a continuously cropped banana garden [75], and its abundance decrease could be attributed to soil pH change (Table 1). In the biochar-treated plots, there were also enhanced abundances of *Ilyonectria* and *Plectosphaerella*, both belonging to pathogenic fungi potentially causing root diseases [76, 77]. Accompanying this change, there was a significant increase in the relative abundance of the phylum *Cadophora*, which potentially forms mycorrhizae with host plants [78] and of *Pseudogymnoascus*, which is known to be a potential biocontrol [79]. Liu et al. [77] noted a see-saw-like change in a ginseng garden with and without root disease roots in abundance of *Ilyonectria* versus *Mortierella* and *Pseudomonadales* versus *Actinomycetales,* respectively, of fungal and bacterial communities. With improved phylum diversity, biochar could enhance potential fungal antagonism against pathogenic diseases for self-defense [80].

Furthermore, pathogenic species of *Fusarium* spp. showed reduced relative abundance under biochar treatments, while the beneficial fungal species effective for biocontrol [76] exerted increases in abundance only under MB compared to MC (Fig. 3). A decrease in *Penicillium* spp. has been widely reported in continuously cropped *P. ginseng* gardens with the accumulation of soil pathogens [81–83]. In our study, the abundance of *Fusarium* spp.*,* a key soil-borne pathogen causing root rot of ginseng [84], was very significantly and negatively correlated with ginseng root rot disease incidence (Fig. 1a). This is consistent with other observations in ginseng fields [65, 85]. In the rhizosphere of *R. pseudostellariae* under prolonged monoculture [86, 87], an increase in the gene abundance of fungal *Fusarium* spp. was observed in line with a decrease in fungal *Penicillium* spp. and some beneficial bacterial species, such as *Burkholderia* spp.

The change in the percentage of reads of fungi in trophic mode again reflected the fungal community shift with the significance of potential eco-functions. The great enhancement of symbiotrophic AMF, disappearance of dung saprotrophs, and a decrease in “pathotrophic” fungal parasites with biochar treatments (Table 3) revealed a shift of the fungal community toward a better defense or health functional association when considered with trophic mode. Previous work reported similar results for biochar-amended rice paddies compared to nonamended rice paddies [35]. Of course, the change in the relative abundance of dung and wood saprotrophs (Table 3) could be attributable to the materials amended. Additionally, the decrease in the “pathotrophic” group was parallel to the decrease in the relative abundance of fungal phyla potentially causing pathogenic diseases (for example, *Fusarium*, Fig. 3). Interestingly, the increase in the AMF percentage of reads under biochar treatments (Table 3) was much greater than that of 34.1% in a global meta-analysis [88]. Early on, the development of AMF could be accelerated with SOM and nutrient enhancement under organic amendments [89]. AMF could compete with pathogenic fungi for resources rather than for space [90] and improve the disease resistance of their host plants and inhibit pathogen infection, playing an important role in controlling soil-borne diseases [91].

Overall, the fungal community was greatly and positively altered under biochar treatments, with increased phylum diversity along with a shift in the community composition of enhanced beneficial fungal groups/species. Such changes could improve soil biological health and, in turn, plant quality in continuously ginseng-cropped soil. This was also supported by the overall ginseng quality change, whereby the ratio of PPD-type monomers (Rb1, Rb2, Rc, and Rd) to PPT-type monomers (Re, Rg1, and Rf) was relatively lower under WB and MB than under MC in this study. As per Kim et al. [36], such a lower ratio could represent a potentially alleviated defense pressure of ginseng. Thus, ginseng production and ginseng quality were promoted through microbial changes in the continuously cropped ginseng field under biochar treatment. In other words, biochar addition could be a promising tool to tackle plant diseases related to soil-borne pathogens in the continuous production of functional crops.

Following Bonanomi et al. [92] and Viger et al. [93], the findings here could shed light on the potential of crop residue biochar to promote plant defense and thus metabolic functions rather than to enhance the biomass production of functional food crops. In Northeast China, serious soil degradation with continuous cropping has disappointed farmers in sustaining ginseng production despite increasing market demand [94]. The ginseng production area was reduced from over 2000 ha to less than 900 ha, while the total fresh ginseng root yield decreased from approximately 8 thousand tons to less than 3 thousand tons from 2001 to 2008 [94]. Meanwhile, in this area, maize residue amounted to 150–180 Mt from the total maize production area of approximately 16 M ha in 2015. Most of these residues were burnt in the field due to poor valorization, losing carbon but inducing air pollution [95]. Thus, converting maize residue into biochar and applying it to soil could offer a strategic solution to restore degraded soil and enhance soil health through a route of bioeconomic development [96]. As such, a close loop of grain production of cereal maize connected to functional crop production of ginseng could be developed to meet the multigoals by the state policies on “Black soil” protection, cropland improvement, and rural revitalization and carbon neutrality in the future.

## Conclusions

In a field study, the production of ginseng biomass and quality, seriously challenged by soil degradation under continuous cropping, was greatly improved through enhanced biological health and, in turn, rhizosphere microbial manipulation against soil-borne pathogenic disease incidence. While the microbial community structure shifted mainly to fungi, biochar amendment led to the promotion of beneficial microbes and a reduction in pathotrophic fungi aligned with the functional assignment. Furthermore, maize biochar exerted better effects than wood biochar in terms of positive changes in AMF and other beneficial bacterial species. Therefore, soil amendment with maize residue biochar could be a strategic solution to regenerate the soil health and quality production of functional root crops while enhancing soil carbon storage in continuously cropped soils. Our study could shed light on the potential manipulation of the soil microbiome with biochar-enhanced soil health against pathogenic diseases for functional crop production rather than through biological health and plant defense. Long-term field studies of the soil-biochar-root system are urged to explore the mechanical linkage between soil fertility, biological health and food quality changes for medicinal root crops.

## Materials and methods

### Site and soil condition

The experimental site was located in Xiaoshan village (41°23’N, 127°32′E), Fusong County, Baishan Municipality, Jilin Province, China (Fig. S1). The area was situated around Changbai Mountain with a mean annual temperature of 4°C and precipitation of 800 mm from 2015 to 2019 under a cold and humid climate. The soil was an aquic-mollic Alfisol with a clay loam texture formed on weathered basalt under the pine forest. The production of *P. ginseng* returned to 1850 in forest soil but shifted to farmland with continuous cropping. Since the 2010s, following the state’s deforestation ban, ginseng production has been managed in gardens with shelters for rain and sunlight control and with manure addition to maintain soil fertility (Fig. S1). Consequently, ginseng production in the area has been seriously stressed by pathogenic diseases, for which pesticides such as procymidone are conventionally applied and monitored in ginseng products.

A typical ginseng field that had been cropped with ginseng for 3 years under ridged farming was selected from a household ginseng farm. The basic properties of the topsoil (0–15 cm) before the experiment were as follows: pH (H_2_O) of 4.57, bulk density of 1.15 g cm^−3^, organic carbon of 17.88 g kg^−1^, total N of 1.29 g kg^−1^, available P of 13.4 mg kg^−1^ and K of 153.1 mg kg^−1^ as well as a cation exchange capacity of 24.87 cmol kg^−1^.

### Experimental design

In the present experiment, compared with conventional manure compost (MC), which is the conventional practice for household farm ginseng production by local farmers, the ginseng field was amended at 20 t ha^−1^ with wood (WB) and maize (MB) biochar MC is a commercially available swine manure compost provided by Qingdao Diendi Biological Technology Co. Ltd, China. WB was derived from the char residue from a gasifier (pyrolysis temperature within 600–650°C) using waste wood chips provided by Jinhua Institute of Bioeconomy, Zhejiang, China. Meanwhile, MB was produced via pyrolysis of maize residue in a partially oxic vertical kiln at of 350–550°C, provided by Gong Xiao Co. LTD, Shanxi, China. Before field amendment, these materials were air-dried, ground to pass through a 2 mm sieve, and homogenized. The basic physico-chemical properties of the amendment materials are given in detail in Table S1, while the structure and additional properties of the two biochars are provided in Fig. S2 and Table S2, respectively.

### Experiment performance

The field experiment was established in the selected field in April 2018. Before the experiment, all the ginseng plants grown for 3 years were evacuated from the ridges. Subsequently, the soil on a ridge was thoroughly tilled and rake-evened. A treatment plot was then laid out with an area of 8.16 m^2^ (5.1 m × 1.6 m), and a 0.2 m wide buffering strip was arranged between two adjacent treatment plots on a ridge. One week before transplantation of new ginseng seedlings, the required amount of an amendment material was hand-spread onto the plot’ surface and evenly incorporated to a depth of approximately 15 cm with a wooden ranker following a tilling operation. As a conventional practice for plant defense, procymidone was applied to the soil at 90 kg ha^−1^ across the plots prior to the amendment.

One week following amendment, healthy and uniform 3-yr-old ginseng seedlings purchased via Shen Boshi Company, Jilin, China, were transplanted into each plot at a density of 18 plants m^−2^ and kept growing until harvest. For nutrient supply, a mineral compound fertilizer (N-P_2_O_5_-K_2_O, 16–16–16, %) provided by Jilin Bangnong Fertilizer Co. LTD, Jilin, China was applied at 900 kg ha^−1^ per annum, while a biochar compound fertilizer (N-P_2_O_5_-K_2_O, 15–15–10, %), with 9% organic carbon from the blended maize biochar, was applied at 600 kg ha^−1^ per annum with both WB and MB treatments. These fertilizers were carefully hand spread onto the soil surface and mixed into the top layer in mid-April each year, consistently throughout the experiment.

Each treatment was performed in triplicate, and all the individual treatment plots were arranged in a randomized block design (Fig. S3). All the farming performances, including temperature/moisture control, weed control and plant protection, as well as ginseng harvest, were followed by local ginseng farmers and kept consistent across the treatment plots.

### Plant sampling and analysis

In this study, the transplanted ginseng plants were harvested after an additional 2 years of growth in the amendment-treated plots. At harvest on June 13^th^, 2020, all the ginseng plants in a plot were evacuated, and the soil material was removed, rinsed with water and pooled. Of this pooled sample, 5 plants were randomly selected for plant trait measurements. Plant size, diameter and length of root tubers were measured with a Vernier caliper while plant gross fresh biomass measured with an electronic balance in field. For each plot, sampled root tubers of the selected ginseng plants were pooled again, sealed in a plastic bag and shipped to the laboratory in an ice box within 24 h following sampling. At harvest, the plant leaf SPAD value was determined with a portable chlorophyll meter (SPAD 502, Konica Minolta Sensing, Japan), and the leaf area was measured with a leaf area meter (WDY-500A, Beijing, China), but the leaf mass was weighed directly with an electronic balance.

After homogenization, a major portion of a root sample was dried at 75°C to a constant weight in a blast drying oven (DHG-9075AE, Jiecheng Experimental Instrument Co., LTD, Shanghai, China), crashed/chopped, and ground to pass a sieve of 0.25 mm. Following Kim et al. [36], the contents of ginsenoside monomers in ginseng roots were analyzed with liquid chromatography (LC-1100 system, Agilent, Beijing, China), while the content of residual pesticides was determined using solid-phase extraction and GC–MS determination, following Fillion et al. [37]. The detailed protocol for analyzing of ginsenosides and residual pesticides is given in SI 1 and SI 2 of the Supplement Material available online, respectively.

### Soil sampling and analysis

While ginseng was harvested, a rhizosphere soil sample of ginseng was collected for microbial analysis. As per the protocols described by Butler [38], all ginseng roots harvested in a plot were gently hand-shaken to remove attached soil particles, which were collected, pooled and homogenized as a rhizosphere sample. Following ginseng harvest completion, 5 individual subsamples in a treatment plot were randomly collected using a stainless steel shovel and homogenized to obtain a composite bulk topsoil (0–15 cm) sample. All the collected soil samples were immediately sealed in steel stainless cans, placed in an ice box and shipped to the laboratory within 24 hrs. On arrival, the rhizosphere samples were immediately stored in the laboratory at −80°C before microbial DNA extraction.

The fresh bulk soil samples were hand crashed, sieved to pass through a 2-mm sieve, and homogenized. Subsequently, a sample was divided into 2 portions. One portion was air-dried and further divided into 2 subportions, one ground to pass a 0.25 mm sieve and another ground to pass a 0.15 mm sieve, prior to physicochemical analyses [39]. Another portion was stored at 4°C for soil water-stable aggregate size fractionation as per Smith et al. [40], for microbial biomass carbon and nitrogen analysis following Vance et al. [41], and for analysis of soil extracellular enzyme activities (EEAs). In detail, EEAs of α-glucosidase, β-glucosidase, β-xylosidase, cellobiohydrolase, polyphenol oxidase, sucrase, urease and N-acetyl-glucosaminidase were performed following Deforest [42] while those of acid phosphatase, sulfatase and peroxidase followed German et al. [43]. The protocols for soil aggregate size fractionation and soil enzyme activity analysis are described in detail in SI 3 and SI 4 of the Supplement Material available online, respectively.

### DNA extraction, real-time qPCR analysis and Illumina HiSeq sequencing

A fresh sample (0.5x g) of rhizosphere stored at −80°C was extracted to obtain total DNA with a Power Soil™ DNA Isolation Kit (Mo Bio Laboratories Inc., CA, USA) (www.mobio.com). This extraction was performed following the manufacturer’s instructions of the kit, and the concentration and quality of the extracted DNA were assessed using a NanoDrop ND-1000 Spectrophotometer (NanoDrop Technologies, United States). Then, qPCR was performed with 12.5 μL of SYBR premix EX Taq™ (Takara Shuzo, Shiga, Japan) in a total volume of 25 μL containing 10 ng DNA, 0.2 μM of a primer and 0.2 mg mL^−1^ BSA. For bacteria and fungi, triplicate tenfold dilutions of plasmid DNA harboring cloned target genes were used to generate standard curves. A melting curve analysis was carried out following each assay to exclude any amplifications from primer dimers or other artifacts. In this study, the qPCR amplification efficiency was 103% for the bacterial 16S rRNA gene and 98% for the fungal ITS gene, with an R^2^ value >0.99.

Using the Illumina HiSeq platform, both bacterial and fungal community compositions were further explored with sequencing target amplicons. Therein, the primer pair 341F/806R was used to target the V3-V4 region of bacterial 16S rRNA genes, while ITS1F/ITS2R was used to target the fungal ITS region. The PCR products were then subjected to gel electrophoresis in the presence of 2% (w/v) agarose. The obtained bands were purified with the AxyPrep DNA Gel Extraction Kit (Axygen Biosciences, USA) (www.axygenbio.com), and their quantification was performed using the QuantiFluor™-ST (Promega-GloMax Promega QuantiFluor, USA). Hereby, the purified amplicons were concentrated on Illumina HiSeq at an isomolar concentration and sequenced as per the standard protocols detailed in the Supplement Information.

The obtained raw sequences were trimmed with QIIME (Quantitative insights into microbial ecology) software (QIIME Pipeline Version 1.8.0) developed by Caporaso et al. [44] (http://qiime.org/tutorials/tutorial.html) and UPARSE Pipeline developed by Edgar [45] (http://drive5.com/uparse/). In doing this, the sequences were screened for quality by eliminating barcodes, primers and low-quality sequences. The remaining sequences were translated into amino acids with the analysis by the Fun Gene Pipeline. Chimeric sequences and singletons were removed with the UCHIME algorithm. The obtained high-quality sequences were clustered into operational taxonomic units (OTUs) at a 97% similarity cut-off. The BLAST algorithm was used to retrieve the NCBI GenBank database, and the representative sequences of an OTU were classified and identified. Data of the resultant OTUs were input into QIIME software to calculate rarefaction curves and community diversity indices. Based on the OTU data, community functions were predicted using PICRUSt [46] coupled with the KEGG Orthology classification scheme for bacteria and using FUNGuild [47] for fungi.

### Co-occurrence network analysis

OTUs with a relative abundance less than 0.01% were removed to reduce rare OTUs in the dataset. To analyze the preprocessed data and calculate the Spearman correlation and the network indices, the psych and igraph packages were used in the calculation with R software (Version 4.0.0). Following an adjustment by Benjamini–Hochberg’s false discovery rate (Version 0.9.2; https://gephi.org/) [48], only the results with a cutoff at an absolute *r* > 0.6 and a *p* < 0.05 were retained, as per Ji et al. [49] and Mo et al. [50] for further network visualization by the “gephi” interactive platform.

### Data processing, calculation and statistical analysis

Basic soil health (or soil quality in general) was assessed as per the soil quality index (SQI) proposed by Doran and Parkin [51], calculated using the equation:

(1) \begin{align*} BSH={\sum}_{i=1}^n Wi\times Si \end{align*}

where *Wi* is the PC weighting factor and *Si is* the value of the *i*^th^ indicator. The dataset and the weights of the selected indicators are given in SI 5 of the Supplement Material available online.

Ginseng root quality was assessed primarily with the total ginsenoside content, which was calculated as the sum of the individual monomer contents:

(2) \begin{equation*} TGm={\sum}_{i=1}^n Wi\times Gi \end{equation*}

where TGm is the normalized total ginsenoside content, G*i* is the content, and W*i* is the weight factor for medicine concern of the *i*^th^ monomer of the ginsenosides. The key monomers of Rb1, Re and Rg3, which are regulated in ginseng medicine by the EU and the USA, were given a weight factor of 1.5, while the other monomers were given a weight factor of 1.0.

Additionally, pesticide residue content has been considered a negative factor of ginseng quality and is routinely monitored under medicine regulation. Thus, an overall ginseng quality production could be assessed with the fresh root biomass yield and the ginseng quality, positively with total ginsenosides but negatively with the residual pesticides, calculated as:

(3) \begin{equation*} PG={Y}_r\times \left( Gm- TP\right) \end{equation*}

where PG is the ginseng quality production in kg ha^−1^; Y_r_ is the fresh root biomass in t ha^−1^; Gm is the normalized total content of ginsenosides in mg g^-1;^ and TP is the total content in mg g^−1^ of residual pesticides DDT, 666 and procymidone of ginseng root.

Finally, the overall improvement of soil health for ginseng production using organic amendments was tentatively assessed concerning the synergism between the key ecological services provided by soil. These key services were concerned with plant production, carbon sequestration, nutrient conservation, habitat condition, and microbial biomass and diversity. These were quantitatively assessed by ginseng quality production, SOC storage, the soil level of nutrients (N, P, K) and moisture, soil pH, EC and MWD, and SMBN, Chao and Shannon index of species. An overall soil improvement was the mean of these relative changes with biochar treatments compared to conventional manure use. Hereby, no weight factor was assigned for the different ecological services provided by soil.

All data were expressed as the means plus/minus one standard deviation of a treatment plot and processed with Microsoft Excel version 2013. Statistical analysis of variance was performed with ANOVA using SPSS software (Version 20.0). Duncan’s test was used to determine the significance of differences among the means, but Pearson’s test was used to assess the significance of a correlation. A difference among treatments or a correlation between analyzed parameters was defined as significant at *p <* 0.05.

## Acknowledgements

This study was partially supported by the Ministry of Science and Technology of China under a grant number of 2017YFD0200802. Funding for advanced soil research was also provided with the project “Double First-Class Discipline Construction Plan” by the Ministry of Education of China. Additional in-kind financial support was obtained from the Jiangsu Collaborative Innovation Center for Solid Organic Waste Resource Utilization, China.

## Author contribution

CL, experiment, sample analysis, data processing and MS drafting; RX, experiment performance and sample analysis; MT, XC and BZ, field experiment, sampling and data collection; XL, RB, LY, JZ, KC and XZ, experiment and data inspection; SJ, MD and LL, supervision and data analysis; SS, experiment design and data interpretation; GP, experiment design, data inspection and analysis, MS editing.

## Data availability

The authors confirm that the experimental data are accessible via the main text and the supplemental material.

## Conflicts of interest

All authors declare no conflicts of interest.

## Supplementary data

Supplementary data is available at *Horticulture Research* online.

## Supplementary Material

Web_Material_uhac108
