# Supplementary material for: Improved ginseng production under continuous cropping through soil health reinforcement and rhizosphere microbial manipulation with biochar: a field study of Panax ginseng from Northeast China
Source: Hortic Res. 2022 May 17;9:uhac108. doi: 10.1093/hr/uhac108 (PMC9273955; doi:10.1093/hr/uhac108)
Supplement: Web_Material_uhac108 [file web_material_uhac108.zip › Language Editing Certificate.pdf]

ELSEVIER

Certificate of Elsevier Language Editing Services

The following article was edited by Elsevier Language Editing Services:

"Improved ginseng production under continuous cropping through soil health reinforcement and rhizosphere microbial manipulation with biochar: A field study of Panax ginseng from Northeast China"

Authored by:

Cheng Liu

Date: 27-Mar-2022
Serial number: LE-235657-BDD424D6411F
