# Supplementary material for: Improved ginseng production under continuous cropping through soil health reinforcement and rhizosphere microbial manipulation with biochar: a field study of Panax ginseng from Northeast China
Source: Hortic Res. 2022 May 17;9:uhac108. doi: 10.1093/hr/uhac108 (PMC9273955; doi:10.1093/hr/uhac108)
Supplement: Web_Material_uhac108 [file web_material_uhac108.zip › Supplement data avaliable online 0402.doc]

**Supplement Material available online**

**MS title:** Improved ginseng production under continuous cropping through soil health reinforcement and rhizosphere microbial manipulation with biochar: A field study of *Panax ginseng* from Northeast China

**Authors:** Cheng Liu^a,b^, Rong Xia^a^, Man Tang^a^, Xue Chen^a^, Bin Zhong^a^, Xiaoyu Liu^a,b^, Rongjun Bian^a,b^, Li Yang^c^, Jufeng Zheng^a,b^, Kun Cheng^a,b^, Xuhui Zhang^a,b^, Marios Drosos^a,b^, Lianqing Li^a,b^, Shengdao Shan^d^, Stephen Joseph^a,e^, Genxing Pan^a,b *^

**Author’s affiliations:**

^a^ Institute of Resource, Ecosystem and Environment of Agriculture, and department of Soil Science, Nanjing Agricultural University, Nanjing 210095, Jiangsu, China

^b^ Jiangsu Collaborative Innovation Center for Solid Organic Waste Resource Utilization, Nanjing Agricultural University, 1 Weigang, Nanjing 210095, China

^c^ College of Chinese Medicinal Materials, Jilin Agricultural University, 28888 Xincheng Street, Changchun 130118 China

^d^ Key Laboratory of Recycling and Eco-treatment of Waste Biomass of Zhejiang Province, Zhejiang University of Science and Technology, Hangzhou 310023, China

^e^ School of Materials Science and Engineering, University of New South Wales, Sydney, New South Wales 2052, Australia

**Corresponding author: Genxing Pan (G. Pan)**

**Email:** [**gxpan@njau.edu.cn**](mailto:gxpan@njau.edu.cn)

**Contents in this file:**

**Supplement Information: 5**

**SI 1.** Determination of ginsenoside content of ginseng root samples

**SI 2.** Determination of residual pesticide contents of ginseng root samples

**SI 3.** Protocol of **s**oil aggregate size fractions separation

**SI 4.** Analysis of soil extracellular enzyme activities

**SI 5.** Prediction of basic soil health index (BSH)

**Supplement Figures: 9**

**Fig. S1** Site location of the experiment in Fusong County, Jilin Province, Northeast China

**Fig. S2** Scanning electron microscopy image of wood chip biochar (Left) and maize straw biochar (Right).

**Fig. S3** Treatment plots layout with ginseng planting ridges in the ginseng farm.

**Fig. S4** Photo images of ginseng growth observed at harvest at 2 years following amendment at 20 t ha^-1^ in the continuously cropped ginseng farm.

**Fig. S5** Shannon index of microbial diversity at phylum level of ginseng rhizosphere sampled in harvest at 2 years following amendment at 20 t ha^-1^ in the continuously cropped ginseng farm.

**Fig. S6** Pearson correlations of root rot disease incidence to relative abundance of *Fusarium* spp.

**Fig. S7** Relationships of microbial abundance and alpha diversity with soil properties (top) and Redundancy analysis (RDA) of bacterial and fungal community changes with soil properties (bottom).

**Fig. S8** Functional traits of bacterial community on KEGG pathway (KO tier 2) at DNA level of rhizosphere soil sampled in harvest at 2 years following amendment at 20 t ha^-1^ with biochar (BA, average of WB and MB) relative to with manure (no biochar, NB). Left panel, relative abundance of different functions of both fields; Right panel, size of the proportion differences in the BA soils from the NB soil at 95% confidence interval, with the significance at *p* < 0.05. MC, manure compost; WB, wood biochar; MB, maize biochar.

**Fig. S9** A systematic diagram showing overall soil health improvement, assessed with the major soil functions and ecological services of ginseng quality production, carbon sequestration, nutrient conservation and availability, habitat condition and microbiome biomass and diversity, in the continuously cropped soil under amendment at 20 t ha^-1^ with WB and MB relative to MC. MC, manure compost; WB, wood biochar; MB, maize biochar.

**Supplement Tables: 9**

**Table S1** Basic properties of amendment materials and fertilizer used in the experiment.

**Table S2** Proximate analysis of the two types of biochar used in the field experiment

**Table S3** Growth traits of transplanted ginseng plants observed in harvest at 2 years following amendment at 20t ha-1 in the continuously cropped ginseng farm.

**Table S4** Content (g kg^-1^) of total and individual monomers of ginsenosides of harvested ginseng roots at 2 years following amendment at 20t ha^-1^ in the continuously cropped ginseng farm.

**Table S5** Concentration of toxic pesticide residues of ginseng roots harvested at 2 years following amendment at 20t ha^-1^ in the continuously cropped ginseng farm.

**Table S6** Bacterial and fungal richness (Chao1) and diversity (Shannon index) of ginseng rhizosphere sampled in harvest at 2 years following amendment at 20t ha^-1^ in the continuously cropped ginseng farm.

**Table S7** Data of soil property used for soil health assessment

**Table S8** Topological properties of co-occurrence network for rhizosphere soil sampled in harvest at 2 years following amendment at 20 t ha^-1^ in the continuously cropped ginseng farm.

**Table S9** Soil quality index (SQI) and indicates of root production and nutrient economic efficiency under the treatments

**SI 1. Determination of ginsenoside content of ginseng root samples**

In performance, a small portion of a fresh ginseng root tuber sample was freeze-dried, ground to pass a 150-mesh sieve. Subsequently, a sample of 1.0 g was extracted with 70% CH_3_OH in a glass flask. The suspension was ultrasonicated at 25 °C for 30 min and then centrifuged at 4000 rpm for 15 min. The supernatant was filtered through a Sep-Pak C18 cartridge (Waters Corp., Milford, MA, USA) and then through a 0.45 μL microfilter. Content of total ginsenosides and the monomers were determined with liquid chromatography (LC-1100 system, Agilent, Beijing, China). Chromatographic separations were conducted on a Halo® RP - Amide column (4.6 × 250 mm, 5.0 μmat, 25 °C). The mobile phase was established with an eluent (acetonitrile: deionized Water, 30:70, v/v), and the flow rate was 1 mL min^-1^. UV detection was conducted at 203 nm, and the injected volume was 20 μL. Standard ginsenosides were provided by the Institute for Drug and Biological Products, China. A concentration of a ginsenoside was quantified as per the relative peak area compared to the internal standard. With this protocol, concentration of totally 12 monomers could be derived, as seen in Table S4. As regulated in EU and USA medicine, Rb1, Re and Rg3 from the total 12 monomers were considered as of key importance for medical use.

**Reference:**

Kim G-S, Lee S-E, Noh H-J, et al (2012) Effects of natural bioactive products on the growth and ginsenoside contents of panax ginseng cultured in an aeroponic system. J Ginseng Res 36:430–441. <https://doi.org/10.5142/jgr.2012.36.4.430>.

**SI 2. Determination of residual pesticide contents of ginseng root samples.**

In detail, a root sample portion was freezing-dried and sieved to pass through a 100-mesh. Subsequently, weigh 1.50 g of this sample into a 50 mL plastic centrifuge tube, add 10 mL of ultrapure water, mix well and place for 2 h. Then, 15 mL of acetonitrile (containing 1% acetic acid) were added to the tube and shaken for 1 min, followed by addition of 1 g of anhydrous magnesium sulfate and 4 g of sodium chloride and subsequent vigorous shaking for 1 min. After centrifugation at 4000 rpm for 10 min, 12 mL of the supernatant was transferred to a 50 mL plastic centrifuge tube, which was steam-swirled to concentrate until nearly dry. Again, 10mL of cyclohexane-ethyl acetate (1:1, v/v) were added to the tube and shaken up, and the contents transferred to another plastic centrifuge tube preloaded with 1 g anhydrous sodium sulfate. The tube was placed for 1h and then vortexed for 1 min and centrifuged again at 2000 rpm for 5 min. 5 mL of the supernatant was transferred to a test tube and steam swirl to concentrate until nearly dry. Then, 1mL of acetonitrile was added to the test tube and transferred to solid phase extraction (SPE) column of flururi silica. The column was washed with n-hexane and the eluent was discarded, before placing the condensed extract in the column and eluting with acetone and n-hexane (1:1, v/v). The eluent was collected and condensed to near dryness, before dissolving in 1 mL of n-hexane. Finally, the extract was passed through a 0.22-μm organic membrane and injected into a GC-MS system to analyze.

GC-MS analysis was performed using a gas chromatography (GC, Agilent 7890B, USA) coupled to a quadrupole mass selective detector (MSD, Agilent 5977B, USA). Analytes were separated by an HP-5MS fused-silica capillary column (30 m × 0.25 mm id, 0.25 μm thickness), using helium as the carrier gas. A sample of 1μL was injected with an Agilent G4513A auto-sampler under splitless condition (temperature hold at 40 °C for 1 min, increased from 40 to 240 °C at a rate of 5 °C min^-1^, from 240 °C to 300 °C at a rate of 12 min^-1^, hold at 300 °C for 6 min), the mass spectrometer was operated in the electron impact (EI) mode at 70 eV ionization energy and acquisition was performed on single ion monitoring (SIM) at the molecular ion of each pesticide at the windows corresponding to the elution region of the target pesticide. Data were acquired and processed with the Agilent Mass Hunter Workstation GC/MS Acquisition and Quantitative Analysis 10.0 software.

**Reference:**

Fillion, J., Sauvé, F. & Selwyn, J. Multiresidue Method for the Determination of Residues of 251 Pesticides in Fruits and Vegetables by Gas Chromatography/Mass Spectrometry and Liquid Chromatography with Fluorescence Detection. J. AOAC Int. 83, 698–713 (2000).

**SI 3.** **Soil aggregate size fraction separation**

In detail, for water-stable soil aggregate size fractionation, a 300-g subsample of the field bulk topsoil sample submerged for 10 min was gently placed onto a 2000 µm nylon sieve in water under a steel basin, which was moved up and down 3 cm to the water table until all soil material passed through the 2000 µm sieve. Plant debris and gravels not passing through the sieve were collected for the calculation of final recovery of the fractionation procedure. The soil suspension passed through the 2000 µm sieve was completely poured onto a 250 µm sieve placed in another basin, and manually moved the sieve up and down 3 cm to the water table with 150 repetitions. The soil material remaining on the sieve were collected as macroaggregates in size of 2000-250 µm. The soil suspension passed through the sieve was again completely poured onto a 53 µm sieve placed in another basin, which was manually moved up and down 3 cm to the water table with 250 repetitions. The soil material remaining on this sieve were collected as microaggregates in size of 250-53μm. Subsequently, the suspension passed through the 53 µm sieve was all transferred to 500-ml plastic centrifuge bottles, which were centrifuged at 4200 rpm for 36 min, according to Stocks law. The residues in all tubes were collected as the fine particles. Finally, all the fractions obtained by wet-sieving and centrifuging were freeze-dried and stored at room temperature prior to analysis.

**References:**

Smith, A.P., Marín-Spiotta, E., de Graaff, MA., Balser, T.C., (2014) Microbial community structure varies across soil organic matter aggregate pools during tropical land cover change. Soil Biol Biochem 77, 292–303. https://doi.org/10.1016/j.soilbio.2014.05.030

**SI 4.** **Analysis of soil extracellular enzyme activities**

The activities of α-glucosidase, β-glucosidase, β-xylosidase, cellobiohydrolase, acid phosphatase, sulfatase and N-acetyl- glucosaminidase were meausred as follows. 200 μl of a homogenized soil slurry and 50μl of 200-μM MUB-linked substrate were dispensed into a 96-well and 300μl microplate of black polystyrene. A substrate plate in six replicates were incubated in dark at 25 °C for 3h and measured with Spectro Max M5 (Molecular Devices, Inc., USA) using 365-nm excitation and 450-nm emission filters. Negative control and MUB standard ranging from 0 to 100μM were prepared in triplicates for each sample to compute the extracellular enzyme activities.

Differently, the procedure for measuring activities of polyphenol oxidase and peroxidase is briefly documented as follows. A volume of 1.0 ml of blended soil slurry was pipetted into 1.5 ml PE plastic centrifuge tube (3 replicates). To the tube was added 250 μl L-DOPA (25 mM) for phenol oxidase analysis and 250 μl L-DOPA and 50 μl 0.3% H_2_O_2_ solution for peroxidase analysis, with a 250 μl sodium acetate buffer as the control. The tubes were incubated waveringly in dark at 30 °C for 18h and subsequently centrifuged at 900 rpm for 4 min. Finally, a 250 μl supernatant of each sample was pipetted into a 96-well clear microplate and the enzyme activity was measured with a micro-plate spectrophotometer using the absorbance at 460 nm.

**References:**

De Forest JL (2009) The influence of time, storage temperature, and substrate age on potential soil enzyme activity in acidic forest soils using MUB-linked substrates and L-DOPA. Soil Biol Biochem 41,1180-1186. [doi:10.1016/j.soilbio.2009.02.029](https://doi.org/10.1016/j.soilbio.2009.02.029).

German DP, Weintraub MN, Grandy AS, et al (2011) Optimization of hydrolytic and oxidative enzyme methods for ecosystem studies. Soil Biol Biochem 43: 1387–1397. <https://doi.org/10.1016/j.soilbio.2011.03.017>.

**SI 5. Prediction of basic soil health index (BSH)**

Briefly, the soil properties were grouped into a small set using principal component analysis (PCA) (Yao et al. 2013). Firstly, nonparametric statistical analysis (Kruskal–Wallis χ2) was conducted to identify indicators with significant differences, and only variables having significant differences (*p* < 0.05) were chosen for PCA (Andrews and Carroll 2001). Then, only the PCs with eigenvalues ≥1 were examined during the PCA process (Brejda et al. 2000) due to the principal components (PCs) with high eigenvalues and variables with high factor loading best represent soil properties. Within each PC, the factor loading of each variable represents its contribution to the PC. Indicators receiving weighted loading values within 10% of the highest weighted loading were selected for the MDS. Each PC was considered in the MDS if the two considered values were not correlated (r < 0.60) when more than one variable was retained in a PC (Andrews et al. 2002). While, only the variable with the highest correlation sum was selected for the MDS among well-correlated variables within a PC (Andrews and Carroll 2001). After selecting the MDS, as each indicator unit is different, the standard scoring function (SSF) is used to calculate each indicator as a standard value (range: 0 ~ 1). Finally, the value of BSH was calculated using the integrated quality index equation (Doran and Parkin 1994).

**References**

Andrews SS, Carroll CR (2001) Designing a soil quality assessment tool for sustainable agroecosystem management. Ecol Appl 11:1573-1585.

Andrews SS, Mitchell JP, Mancinelli R, Karlen DL, Hartz TK, Horwath WR, Pettygrove GS, Scow KM, Munk DS (2002) On-farm assessment of soil quality in California's central valley. Agron J 94:12-23.

Doran JW, Parkin TB (2015) Defining and Assessing Soil Quality. In: Doran JW, Coleman DC, Bezdicek DF, Stewart BA (eds) SSSA Special Publications. Soil Science Society of America and American Society of Agronomy, Madison, WI, USA, pp 1–21.

Yao R, Yang J, Gao P, Zhang J, Jin W (2013) Determining minimum data set for soil quality assessment of typical salt-affected farmland in the coastal reclamation area. Soil Till Res 128:137-148.

**Fig. S1**

**Fig. S2**

**Fig. S3**

**Fig. S4**

b

b

b

c

a

a

**Fig. S5**

**Fig. S6**

**Fig. S7**

**Fig. S8**

**Fig. S9**

**Table S1** Basic properties of amendment materials and fertilizer used in the experiment

| Material | pH  (H_2_O) | Org. C  (g kg^-1^) | Total N  (g kg^-1^) | DOC  (g kg^-1^) | Total P  (g kg^-1^) | Total K  (g kg^-1^) |
| --- | --- | --- | --- | --- | --- | --- |
| MC | 7.73 | 302.30 | 9.30 | 15.22 | 13.40 | 15.60 |
| WB | 10.15 | 601.92 | 2.97 | 2.85 | 3.08 | 7.22 |
| MB | 9.95 | 463.63 | 6.78 | 32.30 | 5.55 | 25.71 |
| MCF | 7.05 | 60.00 | 160.00 | *n.m* | 65.32 | 132.77 |
| BCF | 5.00 | 150.01 | 137.30 | 2.82 | 61.24 | 118.80 |

MC, manure compost; WB, wood biochar; MB, maize biochar; MCF, mineral compound fertilizer; BCF, biochar compound fertilizer. *n.m*., not measured.

**Table S2** Proximate analysis of the two types of biochar used in the field experiment.

| Biochar | LHV | Ash | SA | P.V. | CEC | DOC | C | H | O | N |
| --- | --- | --- | --- | --- | --- | --- | --- | --- | --- | --- |
|  | (MJ kg^-1^) | (%) | (m^2^ g^-1^) | (cm^3^ g^-1^) | (cmol kg^-1^) | (g kg^-1^) | (%) | (%) | (%) | (%) |
| WB | 34.05 | 7.82 | 6.17 | 0.030 | 10.02 | 2.84 | 75.60 | 6.42 | 14.46 | 1.13 |
| MB | 15.91 | 19.84 | 14.50 | 0.029 | 14.50 | 3.32 | 61.13 | 2.91 | 34.26 | 1.71 |

LHV, lower heat value; SA, surface area; P.V., pore volume; CEC, cation exchange capacity; DOC, dissolvable organic carbon. WB and MB, biochar pyrolyzed respectively of wood chips and maize residue as per conditions described in MM text Section of the manuscript.

**Table S****3** Growth traits of transplanted ginseng plants observed in harvest at 2 years following amendment at 20t ha^-1^ in the continuously cropped ginseng farm

| Treat-  ment | Leaf area  (mm^2^ per leaf) | Leaf weight  (g per leaf) | Leaf  SPAD | Stem width  (mm) |
| --- | --- | --- | --- | --- |
| MC | 22.39±1.65b | 0.29±0.06b | 24.06±2.11b | 3.94±0.23a |
| WB | 23.74±1.87b | 0.29±0.03b | 32.08±0.82a | 4.50±0.74a |
| MB | 30.25±4.51a | 0.43±0.09a | 30.33±1.56a | 4.26±0.31a |

MC, amendment of manure compost at 20 t ha^-1^; WB and MB, amendment of wood and maize biochar respectively at 20 t ha^-1^. Different letters in a single column indicated significant difference among treatments at *p* < 0.05.

**Table S4** Content (g kg^-1^) of total and individual monomers of ginsenosides of harvested ginseng roots at 2 years following amendment at 20 t ha^-1^ in the continuously cropped ginseng farm

| Treat-  ment | Total  ginsenoside | Monomer of ginsenosides | | | | | | | | | |
| --- | --- | --- | --- | --- | --- | --- | --- | --- | --- | --- | --- |
|  |  | Rb1 | Rg1 | Re | Rc | Rd | Rf | Rb2 | Rb3 | Rg3 | Rh2 |
| MC | 13.90±1.02b | 2.70±0.37a | 2.65±0.10b | 2.93±0.04a | 1.48±0.10a | 0.75±0.03a | 0.74±0.11a | 0.65±0.09b | 0.49±0.03a | 0.73±0.03a | 0.78±0.12a |
| WB | 13.02±0.60b | 3.03±0.18a | 2.78±0.08ab | 2.99±0.08a | 1.16±0.07b | 0.63±0.03b | 0.42±0.06b | 0.67±0.04b | 0.18±0.02c | 0.76±0.03a | 0.40±0.01b |
| MB | 15.24±0.82a | 2.94±0.20a | 2.91±0.03a | 3.04±0.07a | 1.54±0.15a | 0.75±0.09a | 0.81±0.07a | 1.21±0.10a | 0.41±0.03b | 0.77±0.02a | 0.86±0.06a |

Arranged in abundance order with which the monomers of Rb1, Rg1 and Re were the three key monomers concerned in medicine regulations by EU, USA and China. MC, amendment of manure compost at 20 t ha^-1^; WB and MB, amendment of wood and maize biochar respectively at 20 t ha^-1^. Different letters in a single column indicated significant difference among treatments at *p* < 0.05.

**Table S5** Concentration of toxic pesticide residues of ginseng roots harvested at 2 years following amendment at 20t ha^-1^ in the continuously cropped ginseng farm

| Treat-  ment | α-666  (mg kg^-1^) | p, p'-DDE  (mg kg^-1^) | γ-666  (µg kg^-1^) | δ-666  (µg kg^-1^) | β-666  (µg kg^-1^) | O, P'-DDT  (µg kg^-1^) | P, P'-DDD  (µg kg^-1^) | P, P'-DDT  (µg kg^-1^) | PCM  (µg kg^-1^) | PCNB  (µg kg^-1^) |
| --- | --- | --- | --- | --- | --- | --- | --- | --- | --- | --- |
| MC | 2.24±0.15a | 15.40±3.71a | 16.90±0.86a | 37.18±3.00a | 37.68±3.66a | 47.00±10.90a | 16.30±5.80a | 18.05±2.41a | 439.9±86.5a | *u.d.* |
| WB | 2.38±0.13a | 12.21±4.32a | 14.86±0.54a | 41.02±2.24a | 41.02±2.24a | 37.67±21.47a | 18.19±4.20a | 18.88±2.67a | 357.3±59.3b | *u.d.* |
| MB | 2.00±0.11b | 11.28±1.92a | 16.18±2.58a | 18.25±5.85b | 37.16±6.12a | 42.24±20.38a | 16.64±1.86a | 18.01±1.33a | 292.5±59.2b | *u.d.* |

PCM, Procymidone; PCNB, Pentachloronitrobenzene. MC, amendment of manure compost at 20 t ha^-1^; WB and MB, amendment of wood and maize biochar respectively at 20 t ha^-1^. *u.d*, undetected. Different letters in a single column indicated significant difference between amendment treatments at *p* < 0.05.

**Table S6** Bacterial and fungal richness (Chao1) and diversity (Shannon index) of ginseng rhizosphere sampled in harvest at 2 years following amendment at 20t ha^-1^ in the continuously cropped ginseng farm.

| Treatment | Bacteria | |  | Fungi | |  |
| --- | --- | --- | --- | --- | --- | --- |
|  | Chao1 | Shannon |  | Chao1 | Shannon | |
| MC | 932.28±10.96b | 5.43±0.09b |  | 354.67±9.11b | 3.59±0.54a | |
| WB | 981.32±13.77a | 5.69±0.03a |  | 531.70±23.72a | 4.01±0.10a | |
| MB | 969.64±7.05a | 5.36±0.05b |  | 520.22±15.80a | 4.06±0.43a | |

MC, amendment of manure compost at 20 t ha^-1^; WB and MB, amendment of wood and maize biochar respectively at 20 t ha^-1^. Different letters in a single column indicated significant difference between amendment treatments at *p* < 0.05.

**Table S7** Data of soil property used for soil health assessment

| Treat-  ment | pH (H_2_O) | BD  (g cm^-3^) | SOC  (g kg^-1^) | TN  (g kg^-1^) | AvP  (mg kg^-1^) | AvK  (mg kg^-1^) | CEC  (cmol kg^-1^) | EC  (μs cm^-1^) | Moisture  (%) | MWD  (µm) | MBC  (mg kg^-1^) | MBN  (mg kg^-1^) |
| --- | --- | --- | --- | --- | --- | --- | --- | --- | --- | --- | --- | --- |
| MC | 4.45 | 1.07 | 31.68 | 1.36 | 15.73 | 345.83 | 22.60 | 67.20 | 11.48 | 393.39 | 95.46 | 5.80 |
| MC | 4.34 | 1.09 | 34.62 | 1.46 | 17.65 | 351.04 | 22.29 | 84.90 | 10.44 | 409.45 | 99.62 | 6.57 |
| MC | 4.55 | 1.10 | 30.87 | 1.33 | 20.33 | 326.43 | 23.80 | 72.40 | 11.16 | 392.95 | 101.50 | 6.39 |
| WB | 4.85 | 1.03 | 37.25 | 1.48 | 31.46 | 378.69 | 21.46 | 24.20 | 12.06 | 427.67 | 113.12 | 7.78 |
| WB | 4.81 | 1.01 | 39.83 | 1.39 | 30.69 | 372.17 | 25.10 | 22.70 | 12.78 | 502.66 | 116.05 | 9.30 |
| WB | 4.68 | 1.01 | 35.28 | 1.43 | 34.14 | 370.26 | 22.55 | 22.10 | 13.86 | 437.01 | 113.11 | 10.74 |
| MB | 4.61 | 1.03 | 47.57 | 1.15 | 41.43 | 485.42 | 25.83 | 35.40 | 14.80 | 443.18 | 109.03 | 8.34 |
| MB | 4.66 | 0.92 | 44.77 | 1.73 | 40.28 | 492.93 | 25.42 | 33.40 | 15.84 | 428.61 | 124.00 | 14.77 |
| MB | 4.68 | 0.96 | 48.86 | 1.43 | 44.11 | 493.75 | 27.40 | 36.50 | 16.84 | 429.32 | 110.88 | 10.05 |

SQI, soil quality index; BD, bulk density; SOC, soil organic matter; TN, total nitrogen, AvP, available phosphorus; AvK, available potassium; CEC, cation exchange capacity; EC, electrical conductivity; MWD, mean weight diameter; MBC, microbial biomass carbon; MBN, microbial biomass nitrogen; MC, amendment of manure compost at 20 t ha^-1^; WB and MB, amendment of wood and maize biochar respectively at 20 t ha^-1^.

**Table S7** Data of soil property used for soil health assessment (continued, soil extracellular enzyme activity)

| Treat-  ment | AG  (nmol g^-1^ h^-1^) | BG  (nmol g^-1^ h^-1^) | XYL  (nmol g^-1^ h^-1^) | CB  (nmol g^-1^ h^-1^) | NAG  (nmol g^-1^ h^-1^) | ACP  (nmol g^-1^ h^-1^) | SF  (nmol g^-1^ h^-1^) | PHO  (µmol g^-1^ h^-1^) | PEO  (µmol g^-1^ h^-1^) | UR  (mg g^-1^) | SU  (mg g^-1^) |
| --- | --- | --- | --- | --- | --- | --- | --- | --- | --- | --- | --- |
| MC | 5.64 | 137.16 | 18.46 | 26.02 | 32.32 | 175.81 | 8.02 | 0.29 | 4.64 | 0.03 | 24.93 |
| MC | 5.25 | 138.43 | 21.24 | 26.58 | 29.37 | 174.22 | 8.26 | 0.29 | 4.88 | 0.03 | 27.95 |
| MC | 5.62 | 134.95 | 18.78 | 24.30 | 27.46 | 177.25 | 9.82 | 0.27 | 4.68 | 0.03 | 24.46 |
| WB | 5.54 | 112.23 | 15.48 | 22.58 | 16.36 | 157.96 | 6.20 | 0.30 | 5.33 | 0.18 | 34.71 |
| WB | 7.48 | 111.87 | 17.94 | 22.37 | 17.53 | 158.56 | 8.53 | 0.30 | 5.89 | 0.18 | 34.33 |
| WB | 6.79 | 115.41 | 16.55 | 23.83 | 19.80 | 159.16 | 9.25 | 0.28 | 5.57 | 0.15 | 32.41 |
| MB | 6.81 | 122.16 | 23.24 | 18.66 | 14.44 | 167.95 | 17.44 | 0.29 | 6.18 | 0.19 | 37.22 |
| MB | 8.00 | 123.44 | 25.58 | 20.20 | 16.90 | 166.84 | 19.95 | 0.29 | 6.35 | 0.18 | 36.79 |
| MB | 6.14 | 124.52 | 25.00 | 21.39 | 13.06 | 166.51 | 20.50 | 0.30 | 6.35 | 0.18 | 35.05 |

AG, α-glucosidase; BG, β-glucosidase; XYL, β-xylosidase; CB, cellobiohydrolase; NAG, N-acetyl glucosaminidase; ACP, acid phosphatase; SF, sulfatase; PHO, phenol oxidase; PEO, peroxidase; UR, urease and SU, sucrase. MC, amendment of manure compost at 20 t ha^-1^; WB and MB, amendment of wood and maize biochar respectively at 20 t ha^-1^.

**Table S8** Topological properties of co-occurrence network for rhizosphere soil sampled in harvest at 2 years following amendment at 20 t ha^-1^ in the continuously cropped ginseng farm.

| Microbes | Treatment | Positive correlation | Negative correlation | Nodes | Edges | Average degree | Average path length | Average clustering coefficient | Modularity |
| --- | --- | --- | --- | --- | --- | --- | --- | --- | --- |
| Bacteria | MC | 46.48% | 53.52% | 181 | 398 | 4.398 | 3.78 | 0.097 | 0.527 |
|  | WB | 48.28% | 51.72% | 202 | 435 | 4.307 | 3.93 | 0.098 | 0.553 |
|  | MB | 48.78% | 51.22% | 220 | 533 | 4.845 | 3.75 | 0.118 | 0.514 |
| Fungi | MC | 48.15% | 51.85% | 85 | 81 | 1.906 | 4.52 | 0.131 | 0.685 |
|  | WB | 43.18% | 56.82% | 96 | 88 | 1.833 | 5.59 | 0.131 | 0.699 |
|  | MB | 44.83% | 55.17% | 103 | 116 | 2.252 | 5.91 | 0.084 | 0.694 |

MC, manure compost; WB, wood biochar; MB, maize biochar.

**Table S9** Soil quality index (SQI) and indicates of root production and nutrient economic efficiency under the treatments

| Treatment | Edaphic  Soil health | Biochemical  Soil helath | Root  production  (kg ha^-1^) | Ginsenosides  production  (kg ha^-1^) | Ginseng quality  production  (kg ha^-1^) | Economic  efficiency  (Fertilizer) | Economic  efficiency  (Nutrients) |
| --- | --- | --- | --- | --- | --- | --- | --- |
| MC | 0.57±0.01c | 0.72±0.01c | 758.91±42.92c | 10.54±0.49c | 10.52±0.48c | 0.04±0.01c | 0.010±0.001b |
| WB | 0.62±0.02b | 0.77±0.02b | 1195.10±160.14b | 15.53±1.85b | 15.52±1.84b | 0.11±0.01b | 0.036±0.004a |
| MB | 0.67±0.01a | 0.88±0.04a | 1508.13±176.24a | 22.96±2.61a | 22.94±2.60a | 0.15±0.02a | 0.032±0.004a |

Estimated with the Equation 2 in the MM section text, with ginseng root yield and ginseng quality concerned positively of ginsenosides contents and negatively of toxic pesticide residual contents. Economic efficiency (Fertilizer) is the ginseng quality production value scaled by total nutrients provided with fertilizers while Economic efficiency (Nutrient) is the ginseng quality production value scaled by total nutrients provided with fertilizers and biochars amended; MC, amendment of manure compost at 20 t ha^-1^; WB and MB, amendment of wood and maize biochar respectively at 20 t ha^-1^. Different letters in a single column indicated differences among treatments at *p* <0.05.
